# Versatile platelets contribute to rejuvenation

**DOI:** 10.1093/lifemedi/lnae018

**Published:** 2024-06-28

**Authors:** John M Perry, Meng Zhao

**Affiliations:** Children’s Mercy Hospital, University of Kansas Medical Center, University of Missouri, Kansas City, MO 64108, United States; Key Laboratory of Stem Cells and Tissue Engineering (Ministry of Education), Zhongshan School of Medicine, Sun Yat-sen University, Guangzhou 510080, China

Aging is manifested by the progressive deterioration of tissues and organs, with associated changes in cellular composition, transcriptional programs, epigenetics, and metabolism. Inherent within the aging process is a waning potential for regeneration and response to injury. Considering that younger organisms typically exhibit superior regenerative and healing capacity, the ability to restore these aspects of youth in aging phenotypes has been a long-sought but elusive goal.

Interestingly, aging phenotypes have been partially reversed in animal models. Heterochronic parabiosis, which involves the creation of a surgically connected circulatory system between aged and young organisms, provides an experimental system to test effects of blood-borne factors in aging. Historically, parabiosis showed that blood from a healthy animal could rescue the lethal effects of irradiation, establishing the foundational idea of stem cells being responsible for regenerating hematopoiesis. Heterochronic parabiosis has been shown to improve aged stem cell function, neurogenesis and cognitive function [1]. While anti-aging cytokines present in young blood have been identified, the specific rejuvenation factors responsible for these improvements continue to be a sought-after discovery.

A recent study by Schroer et al. has explored the role of circulating platelets in brain rejuvenation by mitigating brain inflammation and improving cognitive function [2]. Platelets are generated from bone marrow megakaryocytes, which play a pivotal role in blood coagulation and hemostasis. Platelets also release growth factors, chemokines, various RNAs, and extracellular vesicles, dynamically regulating wound healing and the immune response to sites of injury. Schroer et al. showed that introduction of young platelet factors were capable of effectively abating neuroinflammation in aged mice, suggesting a potential avenue for addressing age-related inflammation and cognitive decline.

**Figure 1.**
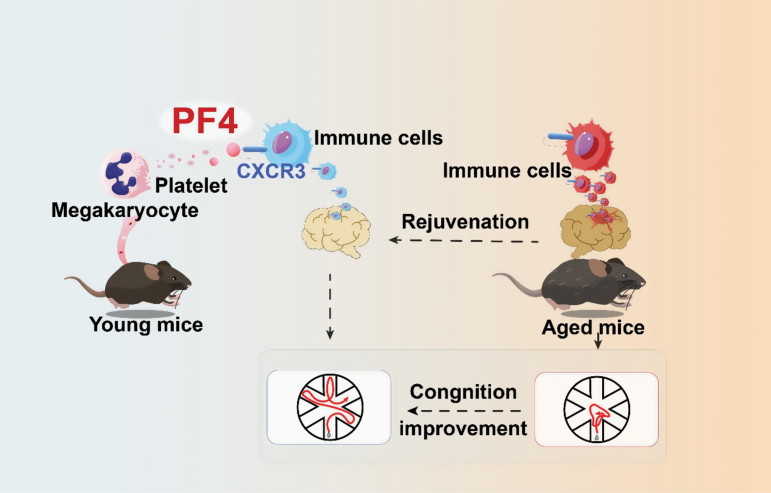
Platelet-derived PF4 enhances cognition in aged mice by rejuvenating their immune system

Megakaryocytes exhibit a wide array of functions, including their involvement in regulating hematopoietic stem cells (HSCs) and orchestrating immune responses against pathogens, achieved through the secretion of a diverse range of factors [3, 4]. Notably, megakaryocytes and platelets serve as a primary source of platelet factor 4 (PF4), also known as CXCL4, to maintain the quiescence of stem cells [3, 4]. Schroer et al. revealed that plasma levels of PF4 experienced a decline in both aged mice and humans. Of particular significance, although PF4 lacks the ability to traverse the blood–brain barrier, the administration of systemic PF4 injections effectively rejuvenated the aged immune system. This intervention mitigated neuroinflammation and subsequently restored the synaptic dynamics and cognitive function within the aged hippocampus. Schroer’s work is complemented by two other studies published by Part et al. and Leiter et al. on the same day [5, 6]. These studies have also explored the role of platelet-derived PF4 in enhancing cognition in aged mice (Fig. 1).

To decipher the downstream mechanism, Schroer et al. have provided evidence supporting the proposition that PF4 could potentially operate in a partial manner through the CXCR3 receptor situated on immune cells. The PF4-CXCR3 axis seems instrumental in rejuvenating the aged immune system and the hippocampus, which indicates that PF4 might partially work through the CXCR3 receptor on immune cells in this restorative effect. It is notable that CXCR2 has also been reported to respond to PF4 on hematopietic stem/progenitor cells [7].

HSCs have been identified as being unusually responsive to young blood in heterochronic parabiosis. Single-cell transcriptomics across aged tissues showed that HSCs serve as a root for the restoration of a young-associated transcriptional regulatory program, resulting in cell state changes across the immune system [8]. Given that megakaryocytes regulate HSCs through PF4 secretion [4] and HSCs serve as the source of all blood, including immune cells, PF4 may ultimately mediate its effects via stem cell regulation. However, the expression of CXCR3 in HSCs is low, as indicated by our unpublished data. This observation suggests the intriguing possibility that PF4 might act on distinct receptors for rejuvenation.

Neurodegenerative diseases currently affect millions throughout the world and, with rapidly growing aging populations, this crisis will only increase without medical intervention. While cell-based therapy has been and should continue to be pursued, the direct delivery of rejuvenating blood-borne factors provides an attractive and perhaps more practical alternative strategy. Continued research similar to that provided by Schroer et al. will provide an avenue for alleviating diseases associated with aging, including the increasing problem of neurodegeneration.

In a collective view, the intricate involvement of megakaryocytes and platelets in both pathogen-triggered immune responses and the inflammatory aspects of aging underscores the imperative need for further investigation. As the association between heightened inflammation and the aging process gains recognition across various organs, the findings open up the tantalizing possibility that PF4 could exert a broader influence. This influence might encompass a reduction in systemic aging-associated inflammation, accompanied by the revival of other tissue functionalities during aging or even an extension of overall lifespan.
